# Diagnostic performance of the urinary canine calgranulins in dogs with lower urinary or urogenital tract carcinoma

**DOI:** 10.1186/s12917-017-1032-5

**Published:** 2017-04-21

**Authors:** Romy M. Heilmann, Elizabeth A. McNiel, Niels Grützner, David J. Lanerie, Jan S. Suchodolski, Jörg M. Steiner

**Affiliations:** 1 College of Veterinary Medicine, University of Leipzig, An den Tierkliniken 23, DE-04103 Leipzig, Germany; 2 Gastrointestinal Laboratory, Texas A&M University, TAMU 4474, College Station, TX 77843-4474 USA; 3 Cummings School of Veterinary Medicine, Tufts University, 200 Westboro Rd, North Grafton, MA 01536 USA; 4 College of Veterinary Medicine, Michigan State University, 784 Wilson Rd, East Lansing, MI 48824 USA; 5 Farm Animal Clinic, Vetsuisse Faculty, University of Bern, Bremgartenstrasse 109a, CH-3012 Bern, BE Switzerland

**Keywords:** Biomarker, Calprotectin, Diagnostic accuracy, S100A8/A9, S100A12, Transitional cell carcinoma

## Abstract

**Background:**

Onset of canine transitional cell carcinoma (TCC) and prostatic carcinoma (PCA) is usually insidious with dogs presenting at an advanced stage of the disease. A biomarker that can facilitate early detection of TCC/PCA and improve patient survival would be useful. S100A8/A9 (calgranulin A/B or calprotectin) and S100A12 (calgranulin C) are expressed by cells of the innate immune system and are associated with several inflammatory disorders. S100A8/A9 is also expressed by epithelial cells after malignant transformation and is involved in the regulation of cell proliferation and metastasis. S100A8/A9 is up-regulated in human PCA and TCC, whereas the results for S100A12 have been ambiguous. Also, the urine S100A8/A9-to-S100A12 ratio (uCalR) may have potential as a marker for canine TCC/PCA. Aim of the study was to evaluate the diagnostic accuracy of the urinary S100/calgranulins to detect TCC/PCA in dogs by using data and urine samples from 164 dogs with TCC/PCA, non-neoplastic urinary tract disease, other neoplasms, or urinary tract infections, and 75 healthy controls (nested case-control study). Urine S100A8/A9 and S100A12 (measured by species-specific radioimmunoassays and normalized against urine specific gravity [S100A8/A9_USG_; S100A12_USG_], urine creatinine concentration, and urine protein concentration and the uCalR were compared among the groups of dogs.

**Results:**

S100A8/A9_USG_ had the highest sensitivity (96%) and specificity (66%) to detect TCC/PCA, with specificity reaching 75% after excluding dogs with a urinary tract infection. The uCalR best distinguished dogs with TCC/PCA from dogs with a urinary tract infection (sensitivity: 91%, specificity: 60%). Using a S100A8/A9_USG_ ≥ 109.9 to screen dogs ≥6 years of age for TCC/PCA yielded a negative predictive value of 100%.

**Conclusions:**

S100A8/A9_USG_ and uCalR may have utility for diagnosing TCC/PCA in dogs, and S100A8/A9_USG_ may be a good screening test for canine TCC/PCA.

## Background

Transitional cell carcinoma (TCC) is the most common naturally occurring lower urinary tract malignancy in dogs affecting a number of canine patients [1–3]. Several predisposing factors (including the use of certain drugs, chemicals, or preventive medications, breed, gender, and diet) have been evaluated (reviewed by Fulkerson et al. [3]). Despite many advances in the management of dogs with TCC or prostatic carcinoma (PCA), early detection of these neoplasms is rare, and their onset is usually insidious with dogs presenting at an advanced stage of the disease (20–40% with metastasis) [1–4]. Thus, as with any cancer, a biomarker that can facilitate early detection of TCC/PCA (i.e., cancer screening) and improve patient survival would be a useful tool in clinical practice.

Urinary cytology as a test for TCC/PCA has been demonstrated to have a low sensitivity and specificity [3, 5]. The veterinary bladder tumor antigen (V-BTA) test has been evaluated as a non-invasive screening tool for TCC in dogs and is currently the biomarker with the best diagnostic accuracy for non-invasive TCC diagnosis, but this test was shown to have a high false-positive rate (12–65%) [6–9]. Concentrations of urinary basic fibroblast growth factor was also increased in dogs with bladder cancer and to some extent discriminated dogs with TCC from dogs with a urinary tract infection (UTI) [10], but sensitivity and specificity of urinary basic fibroblast growth factor have not been reported in a larger population of dogs. A recent urine metabolomics study identified a metabolite signature that could discriminate dogs with TCC from healthy control dogs with a sensitivity of 86% and a specificity of 78% [11], and a mass spectrometry analysis of canine urine samples identified a multiplex biomarker model predicting the presence of TCC with 90% diagnostic accuracy [12]. However, the utility of these models as either a screening tool or a diagnostic test for canine TCC has not been evaluated to date.

S100A8/A9 (calgranulin A/B) and S100A12 (calgranulin C) are members of the S100/calgranulin family of Ca^2+^-binding proteins, and both members are expressed by cells of the innate immune response [13]. Aside from its association with several inflammatory diseases [13, 14], the S100A8/A9 protein complex (also referred to as calprotectin) has been shown to be expressed by epithelial cells after malignant transformation [15–18] and to be involved in the regulation of cell proliferation [19] and metastasis [20, 21]. In humans, both S100A9 and S100A8 were found to be up-regulated in PCA and TCC [22–30], whereas studies on the expression of S100A12 in TCC/PCA have yielded ambiguous results [31, 32]. However, S100A12 has not been investigated extensively in cancer patients.

Similar to human urothelial carcinoma, the *S100A8/A9* gene has been shown to harbour an increased frequency of copy number gains in canine urothelial carcinoma [33], and pilot data by our group suggested that the urinary concentration of S100/calgranulins, particularly the S100A8/A9-to-S100A12 ratio (uCalR), may have potential as a diagnostic biomarker for TCC/PCA in dogs [34]. Thus, further evaluation of the urinary S100/calgranulins and uCalR in dogs is needed to determine their specificity for the diagnosis of TCC/PCA. This retrospective case-control study investigated urine S100A8/A9 and S100A12 concentrations as well as the uCalR in dogs with TCC/PCA (treated or treatment-naïve), dogs with other diseases of the urinary tract, dogs with neoplastic diseases not involving the urinary tract, and healthy control dogs. We hypothesized (1) that the concentration of S100/calgranulins in urine specimens is a useful screening test for TCC/PCA and (2) that the uCalR can distinguish dogs with TCC/PCA and UTI with high diagnostic accuracy.

## Methods

### Sampling population

**Fig. 1:**
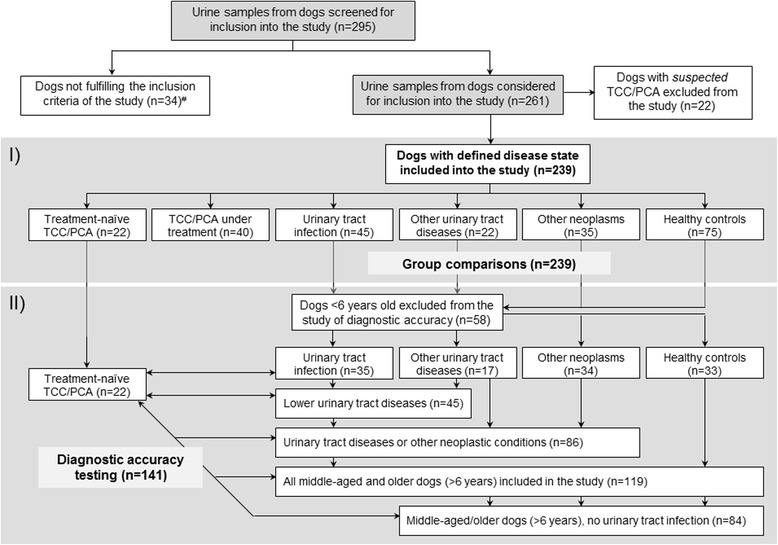
Study flow chart summarizing the group distribution of the 239 dogs included into the study. The two different parts of the study are marked by the grey shaded areas: (i) evaluation of the urinary S100/calgranulins in dogs with a defined disease state; and (ii) evaluation of the diagnostic accuracy of the urinary S100/calgranulins for the diagnosis of transitional cell carcinoma (TCC)/prostatic carcinoma (PCA) in dogs ≥6 years of age (*n* = 141). ^#^e.g., lack of a definitive diagnosis, not possible to categorize in one group (e.g., concurrent non-urinary tract malignancy and urinary tract infection), abnormal urinalysis results in healthy controls, or Leptospirosis suspect (analysis of urine considered as a possible public health concern)

A nested case-control study design was used. Urine samples from dogs with TCC/PCA that were treatment-naïve (*n* = 22) or receiving specific anticancer treatment at the time of sampling (*n* = 40), dogs with non-neoplastic diseases of the urinary tract (*n* = 22), dogs with other neoplasms (*n* = 35), dogs with a urinary tract infection (UTI; *n* = 45), and a group of healthy control dogs (*n* = 75) were used. The study design is summarized in the flow chart (Fig. 1). Cases were recruited at the Veterinary Medical Teaching Hospitals (VMTH) at Texas A&M University (*n* = 99), Michigan State University (*n* = 62) as part of another study [9], and three other North American universities (*n* = 40) as well as from seven Oncology referral practices located in five different US states (*n* = 38). Samples from some of the dogs (9 dogs with TCC/PCA, 9 dogs with a UTI, and 38 healthy dogs) had also been included in the assay validation study [34].

Complete patient information was extracted from the electronic medical records (for all VMTH cases) or a study questionnaire that had to be completed by the owner and/or the attending veterinary oncologist prior to patient enrolment (for all referral practice cases).

### Sample collection and analyses

Urine samples were obtained by cystocentesis, urethral catheterization, or free-catch, and were prepared for analysis of S100A8/A9 and S100A12 as previously described [34]. Briefly, debris was separated from samples by centrifugation for three minutes at 1000 × g, and the supernatants were placed into cryovials and stored frozen at −80 °C until analyses. Urine samples were then thawed and diluted 1:2 in 0.05 M sodium phosphate, 0.02% sodium azide, and 0.5% bovine serum albumin (pH 7.5) [34].

Urine S100A8/A9 and S100A12 were measured in all samples using established and validated species-specific in-house radioimmunoassays [34]. Urine concentrations of S100A8/A9 and S100A12 were normalized against urine specific gravity^1^ (S100A8/A9_USG_ and S100A12_USG_), urine creatinine concentration^2^ (S100A8/A9_Cre_ and S100A12_Cre_), and urine protein concentration^3^ (S100A8/A9_Prot_ and S100A12_Prot_) [34]. In addition, the S100A8/A9-to-S100A12 ratio (uCalR) was calculated for each sample [34].

### Data analyses

Commercial statistical software packages^4^^,^ 5 were used for all statistical analyses. Data were tested for the assumptions of normality and equality of variances using a Shapiro-Wilk and a Brown-Forsythe test, respectively. All parameters (S100A8/A9_USG_, S100A8/A9_Cre_, S100A8/A9_Prot_, S100A12_USG_, S100A12_Cre_, S100A12_Prot_, and uCalR) were tested among the six different groups of dogs using non-parametric multiple- (Kruskal-Wallis test followed by a Dunn’s test for multiple comparisons) or two-group (Mann-Whitney *U* test) comparisons. Summary statistics are reported as medians and interquartile ranges (IQR).

Receiver operating characteristic (ROC) curves were constructed to determine the sensitivities and specificities of normalized urinary S100A8/A9, S100A12, and the uCalR to distinguish dogs with TCC/PCA from the other groups of dogs; the Youden index was used to establish the optimum cut-off values. Positive (PPV) and negative predictive values (NPV) were calculated for the best-performing markers; the prior probability for a diagnosis of TCC/PCA in middle aged to older dogs (defined as ≥6 years of age) was estimated at 0.7% [2], and in dogs older than 6 years after exclusion of a UTI and those with clinical signs of lower urinary tract disease was estimated at 1% and 20%, respectively [8]. Significance was set at *P* < 0.05.

## Results

### Study population

#### Treatment-naïve TCC/PCA dogs

Breeds included Beagle (*n* = 3), Labrador Retriever (*n* = 3), Australian Cattle dog (*n* = 2), Scottish Terrier (*n* = 2), Shetland Sheepdog (*n* = 2), Australian Shepherd dog, Boston Terrier, Chow, Fox Terrier, Lhasa Apso (each *n* = 1), and mixed breed (*n* = 5). The most common tumor location was the urinary bladder (*n* = 15), followed by the urethra (*n* = 7), and the prostate (*n* = 5). The diagnosis was confirmed by histopathologic evaluation of a tissue biopsy (*n* = 7) or cytology (*n* = 15). A urine culture was performed in 12 dogs, 5 (42%) of which were positive for bacterial growth and the majority (80%) of positive samples were growing a single organism (*E. coli*, *Enterococcus* sp., *Pseudomonas aeruginosa*, or *Streptococcus equisimilis*).

#### TCC/PCA dogs under treatment

Breeds included Beagle (*n* = 5), Shetland Sheepdog (*n* = 4), West Highland White Terrier (*n* = 4), Scottish Terrier (*n* = 2), American Spitz, Bichon Frise, English Springer Spaniel, Jack Russell Terrier, Labrador Retriever, Maltese, Miniature Pinscher, Miniature Schnauzer, Papillion, Poodle, Rat Terrier, Schipperke (each *n* = 1), and mixed breed (*n* = 13). Most common tumor site was the urinary bladder (*n* = 33), followed by the prostate (*n* = 7), and the urethra (*n* = 6). Treatment at the time of urine sample collection included non-steroidal anti-inflammatory drugs (NSAID; piroxicam: *n* = 30; carprofen: *n* = 4; firocoxib: *n* = 2; deracoxib: *n* = 1), chemotherapy (mitoxantrone: *n* = 5; cyclophosphamide: *n* = 1), a tyrosine kinase inhibitor (toceranib phosphate: *n* = 3), and/or surgical excision (*n* = 2). The diagnosis had been previously confirmed by histopathology or cytology. A urine culture was performed in 7 dogs, 3 (43%) of which were positive for bacterial growth and a single organism (*E. coli*, *Aerococcus* sp., *Mycoplasma canis*) being isolated in the majority (80%) of the dogs that had a positive urine culture.

#### Dogs with UTI

Breeds included Beagle (*n* = 3), Boston Terrier (*n* = 3), Labrador Retriever (*n* = 3), Pug (*n* = 3), Standard Schnauzer (*n* = 3), Bichon Frise (*n* = 2), Brittany Spaniel (*n* = 2), Dachshund (*n* = 2), Golden Retriever (*n* = 2), Australian Cattle dog, Boxer, Cocker Spaniel, English Bulldog, Fox Terrier, French Bulldog, Great Dane, Husky, Jack Russell Terrier, Lhasa Apso, Miniature Poodle, Miniature Schnauzer, Pembroke Welsh Corgi, Rat Terrier, Rottweiler, Shih Tzu, Swiss Mountain dog (each *n* = 1), and mixed breed (*n* = 5). Urine culture was positive in 44/45 (98%) dogs, and marked bacteriuria but a negative culture was seen in one dog. The isolated bacterial strains were documented in 38 dogs, 6 (16%) of which had a polymicrobial UTI. The most common isolate was *E. coli* (*n* = 21), followed by *Enterococcus* sp. (*n* = 5), *Klebsiella* (*n* = 3), *Proteus mirabilis* (*n* = 6), *Staphylococcus pseudintermedius* (*n* = 2), *Streptococcus canis* (*n* = 2), *Actinobacillus* sp., *Bacillus* sp., *Enterobacter* sp., *Pasteurella* sp., *Pseudomonas aeruginosa* (each *n* = 1). A recurrent UTI was seen in one dog and a resistant UTI in two dogs. One dog had concurrent bilateral ureteral ectopy and one dog had urolithiasis.

#### Dogs with non-neoplastic urinary tract diseases

Breeds included Miniature Schnauzer (*n* = 3), Beagle (*n* = 2), Maltese (*n* = 2), Australian Shepherd, Bichon Frise, Bloodhound, Chihuahua, Dalmatian, Golden Retriever, Labrador Retriever, Miniature Poodle, Standard Schnauzer, Soft Coated Wheaten Terrier, Shih Tzu, West Highland White Terrier, Yorkshire Terrier (each *n* = 1), and mixed breed (*n* = 2). Diseases included urolithiasis (*n* = 11), International Renal Interest Society (IRIS) stage I-IV chronic kidney disease (*n* = 7), detrusor sphincter dyssynergia, polypoid cystitis, urethral stricture, or urethral sphincter mechanism incompetence (each *n* = 1).

#### Dogs with non-urinary tract neoplasia

Breeds included Beagle (*n* = 6), Labrador Retriever (*n* = 5), Shetland Sheepdog (*n* = 3), West Highland White Terrier (*n* = 3), Golden Retriever (*n* = 2), Scottish Terrier (*n* = 2), Basset Hound, Bearded Collie, Boxer, Brittany Spaniel, Cockapoo, German Shorthaired Pointer, Greater Swiss Mountain dog, Rottweiler (each *n* = 1), and mixed breed (*n* = 6). Neoplastic diseases were: lymphoma (*n* = 5), mast cell tumor (*n* = 5), hepatocellular carcinoma (*n* = 4), appendicular osteosarcoma (*n* = 3), undefined adrenal masses (*n* = 2), undefined splenic masses with hemoabdomen (*n* = 2), thymoma (*n* = 2), adrenal carcinoma, hemangiosarcoma, hepatic carcinoma, oral soft tissue sarcoma, oral squamous cell carcinoma, perianal adenoma, plasma cell tumor, pulmonary carcinoma, renal adenocarcinoma, undefined intracranial neoplasia, undefined mandibular neoplasia, and undefined mesenchymal neoplasia (each *n* = 1). Oncologic therapy at the time or prior to urine sample collection for the study included surgery (*n* = 12), chemotherapy (*n* = 8), and/or radiation therapy (*n* = 2).

#### Healthy control dogs

Breeds included West Highland White Terrier (*n* = 7), Beagle (*n* = 4), Labrador Retriever (*n* = 4), Dachshund (*n* = 3), Scottish Terrier (*n* = 3), Shetland Sheepdog (*n* = 3), American Pitbull Terrier (*n* = 2), Giant Schnauzer (*n* = 2), Miniature Schnauzer (*n* = 2), Weimaraner (*n* = 2), Yorkshire Terrier (*n* = 2), Australian Shepherd, Basset Hound, Bichon Frise, Border Collie, Boston Terrier, Boxer, Chihuahua, Chinese Shar Pei, English Springer Spaniel, French Bulldog, Golden Retriever, Great Dane, Neopolitan Mastiff, Portuguese Water dog, Saint Bernard, Standard Poodle, White Shepherd dog (each *n* = 1), and mixed breed (*n* = 17); breed was not documented in 7 dogs. Urinalysis was unremarkable in 64 dogs and was not performed on the same urinalysis in 11 dogs (these dogs were determined to be healthy based on previous diagnostics). Age-related conditions (e.g., degenerative joint disease) not reported to affect the urinary tract were not considered as an exclusion criterion.

### Among group comparisons

**Fig. 2:**
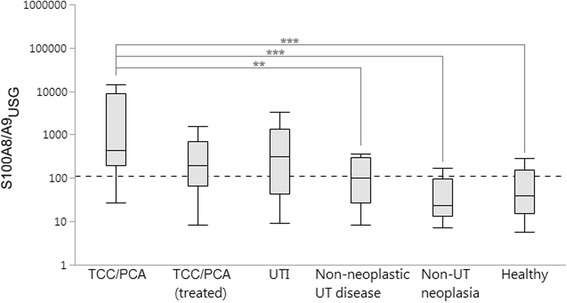
Urinary concentrations of S100A8/A9 normalized against urine specific gravity (S100A8/A9_USG_) were significantly higher in newly diagnosed TCC/PCA dogs compared to dogs with non-neoplastic urinary tract (UT) diseases (*P* = 0.0088), dogs with neoplastic diseases not involving the urinary tract (*P* < 0.0001), and healthy control dogs (*P* < 0.0001); but did not differ from S100A8/A9_USG_ measured in dogs with TCC/PCA undergoing cancer therapy (*P* = 0.2587) or dogs with a bacterial urinary tract infection (UTI; *P* = 0.1763). *Boxes*: interquartile range (IQR); vertical lines within boxes: medians; whiskers: determined by the outermost data points or values computed as (25th quartile – 1.5 × IQR) or (75th quartile +1.5 × IQR); *dashed line*: optimum cut-off concentration (≥109.9) to distinguish dogs with TCC/PCA from those of other groups; **significant difference at *P* < 0.01; ***significant difference at *P* < 0.001

Patient characteristics, urine S100A8/A9 and S100A12 concentrations, and the uCalR are summarized in Table 1. S100A8/A9_USG_ (Fig. 2) and S100A8/A9_Cre_ were numerically higher in dogs with untreated TCC/PCA than in dogs with TCC/PCA under treatment, but both differences did not reach significance (*P* = 0.2587 and 0.3715, respectively). No differences in S100A8/A9_USG_ (Fig. 2) and S100A8/A9_Cre_ were seen between treatment-naïve TCC/PCA dogs and those with a UTI (*P* = 0.1763 and 1.0000, respectively). Compared to treatment-naïve TCC/PCA dogs, urinary S100A12 concentrations were also lower in TCC/PCA dogs undergoing treatment, but the difference was only significant for S100A12_Cre_ (*P* = 0.0416; *P* = 0.0664 for S100A12_USG_), whereas dogs with a UTI had numerically higher (but not significant) S100A12_USG_ and S100A12_Cre_ (both *P* = 1.0000).

**Table 1:** Patient characteristics, normalized urine S100A8/A9 and S100A12 concentrations, and the urine S100A8/A9-to-S100A12 ratio (uCalR) in all dogs included in the study (*n* = 239)

Parameter	TCC/PCA, treatment-naïve	TCC/PCA, under treatment	Bacterial urinary tract infection	Non-neoplastic UT disease	Other, non-UT neoplasia	Healthy control dogs	*P* value^†^
Total number	22	40	45	22	35	75	–
Age^c^, in years median [IQR]	**10.2** ^**A**^ [8.6–12.8]	11.1^A^ [9.5–12.8]	8.8^A^ [5.9–11.5]	10.0^A^ [8.0–11.7]	9.4^A^ [8.4–11.3]	**5.5** ^**B**^ [3.0–8.8]	**<0.0001**
Weight^a^, in kg median [IQR]	18.2^A^ [10.9–28.6]	13.7^A^ [9.8–18.7]	12.7^A^ [8.3–21.5]	9.8^A^ [4.8–16.3]	21.8^A^ [13.7–35.7]	18.1^b, A^ [10.2–26.0]	**0.0006**
Sex male/female	9/13	20/20	12/33	9/13	17/18	40/32^b^	0.0618
S100A8/A9_USG_^c^ median [IQR]	**438.5** ^**A**^ [199.8–8864.1]	196.3^A^ [67.9–687.0]	310.7^A^ [44.3–1384.5]	**100.8** ^**B**^ [28.4–290.1]	**23.5** ^**B**^ [13.4–97.6]	**40.4** ^**B**^ [15.3–145.3]	**<0.0001**
S100A8/A9_Cre_^c^ median [IQR]	**933.6** ^**A**^ [353.8–11,600.2]	445.4^A^ [145.5–1881.4]	956.3^A^ [151.2–4242.0]	**167.8** ^**B**^ [42.4–606.6]	**53.3** ^**B**^ [29.6–225.7]	**23.4** ^**B**^ [4.5–123.6]	**<0.0001**
S100A8/A9_Prot_^c^ Median [IQR]	**201.8** ^**A**^ [43.6–2541.9]	81.7^A^ [36.1–259.0]	176.0^A^ [33.5–637.3]	**37.8** ^**B**^ [6.7–107.3]	**16.4** ^**B**^ [5.0–66.7]	151.5^A^ [69.7–699.7]	**<0.0001**
S100A12_USG_^c^ median [IQR]	**12.4** ^**A**^ [9.1–283.9]	5.0^A^ [2.6–30.6]	44.5^A^ [5.0–308.4]	**2.8** ^**B**^ [1.7–16.8]	**1.9** ^**B**^ [1.3–3.1]	**4.5** ^**B**^ [3.0–7.6]	**<0.0001**
S100A12_Cre_^c^ median [IQR]	**35.2** ^**A**^ [14.7–803.6]	**12.1** ^**B**^ [4.2–61.5]	176.3^A^ [11.2–1323.1]	**4.9** ^**B**^ [2.2–44.5]	**3.9** ^**B**^ [2.7–6.6]	**6.1** ^**B**^ [4.2–10.5]	**<0.0001**
S100A12_Prot_^c^ median [IQR]	**7.5** ^**A**^ [2.4–44.4]	3.0^A^ [1.0–12.7]	41.3^A^ [2.3–129.8]	**2.4** ^**B**^ [0.5–6.7]	**1.6** ^**B**^ [0.2–6.4]	10.9^A^ [5.7–18.4]	**<0.0001**
uCalR^c^ median [IQR]	**14.7** ^**A**^ [10.6–37.7]	29.9^A^ [7.5–71.1]	**6.9** ^**B**^ [2.2–14.0]	14.2^A^ [12.1–36.5]	12.1^A^ [8.5–30.7]	10.6^A^ [5.1–26.6]	**<0.0001**

*TCC* transitional cell carcinoma, *PCA* prostate gland carcinoma, *UT* urinary tract; ^a^no significant differences detected when comparing individual groups with TCC/PCA group; ^b^unknown in 3 dogs; ^c^medians [IQR in brackets] within a row not sharing a common superscript (boldface) are significantly different from treatment-naïve TCC/PCA dogs at *P* < 0.05. ^†^Global *P* value (boldface values indicate a significant difference at *P* < 0.05)

**Fig. 3:**
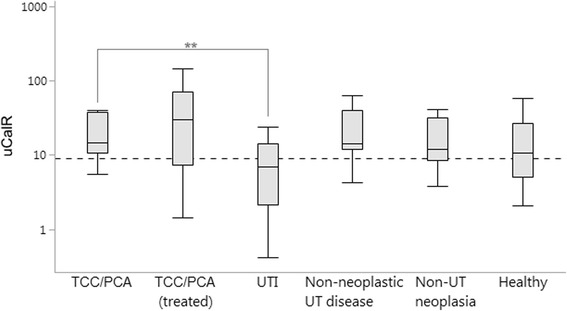
Urinary S100A8/A9-to-S100A12 ratios (uCalR) were significantly lower in dogs diagnosed with a urinary tract infection (UTI) compared to treatment-naïve TCC/PCA dogs (*P* = 0.0014), but did not differ between newly diagnosed TCC/PCA dogs and all other disease groups (all *P* = 1.0000) or healthy control dogs (*P* = 0.3030). *Boxes*: interquartile range (IQR); vertical lines within boxes: medians; whiskers: determined by the outermost data points or values computed as (25th quartile – 1.5 × IQR) or (75th quartile +1.5 × IQR); *dashed line*: optimum cut-off concentration (≥9.1) to distinguish dogs with TCC/PCA from dogs with a UTI; **significant difference at *P* < 0.01

The uCalR did not differ between newly diagnosed TCC/PCA dogs and those undergoing treatment (*P* = 1.0000), but the uCalR was significantly lower in dogs diagnosed with a UTI compared to all other disease groups of dogs: treatment-naïve TCC/PCA dogs (*P* = 0.0014) (Fig. 3), TCC/PCA dogs undergoing treatment (*P* < 0.0001), non-neoplastic urinary tract diseases (*P* = 0.0049), and neoplastic disease not involving the urinary tract (*P* = 0.0364).

S100A8/A9_Prot_ and S100A12_Prot_ were significantly higher in dogs with TCC/PCA than those with non-neoplastic urinary tract diseases (*P* = 0.0079 and 0.0485, respectively) or other neoplasms (*P* < 0.0001 and 0.0056, respectively) but were comparable to those in dogs with a UTI (both *P* = 1.0000) and healthy controls (both *P* = 1.0000).

Comparing urinary canine S100/calgranulin concentrations amongst the same groups of dogs after exclusion of all dogs <6 years of age yielded similar results, with the exception that the difference did not reach significance for S100A12_USG_ in treatment-naïve TCC/PCA dogs compared to dogs with non-neoplastic urinary tract disease (*P* = 0.0574) and healthy control dogs (*P* = 0.0566), for S100A12_Cre_ between treatment-naïve TCC/PCA dogs and those dogs with TCC/PCA under treatment (*P*= 0.1526), and for S100A12_Prot_ between treatment-naïve TCC/PCA dogs and dogs with non-neoplastic urinary tract disease (*P* = 0.2245).

### Within group comparisons

In patients with TCC/PCA, S100A8/A9_USG_, S100A8/A9_Cre_, S100A8/A9_Prot_, S100A12_USG_, S100A12_Cre_, S100A12_Prot_, and uCalR did not differ between dogs with a single tumor site (bladder, urethra, or prostate; *n* = 53) and dogs with more than one of those sites affected (*n* = 9; all *P* > 0.05). This finding was no different in treatment-naïve TCC/PCA dogs (all *P* > 0.05) and those dogs receiving antitumor therapy at the time of sample collection (all *P* > 0.05).

S100A8/A9_Prot_ and S100A12_Prot_ were significantly higher in treatment-naïve TCC/PCA dogs with a positive urine culture (*n* = 5; median [IQR]: 604.8 [392.9–10,353.6] and 39.8 [13.4–870.0], respectively) than dogs without a concurrent UTI (*n* = 7; median [IQR]: 44.5 [32.4–181.2] and 2.5 [1.1–6.1], respectively; both *P* = 0.0230), whereas no differences in S100A8/A9_USG_, S100A8/A9_Cre_, S100A12_USG_, S100A12_Cre_, or uCalR were seen between both groups of dogs (all *P* > 0.05).

S100A8/A9_USG_, S100A8/A9_Cre_, S100A8/A9_Prot_, S100A12_USG_, S100A12_Cre_, S100A12_Prot_, and uCalR did not differ among the three different primary tumor sites in newly diagnosed TCC/PCA dogs (all global *P* > 0.05), whereas S100A8/A9_USG_, S100A12_USG_, S100A12_Cre_, and S100A12_Prot_ were significantly lower with primary involvement of the urinary bladder (*n* = 31; median [IQR]: 161.4 [67.3–361.5], 3.3 [2.3–13.5], 6.6 [3.6–32.9], and 2.0 [0.7–9.5], respectively) than the prostate (*n* = 7; median [IQR]: 1849.6 [563.1–5641.2], 239.6 [10.3–1274.4], 254.0 [18.9–2196.2], and 58.1 [9.2–119.0], respectively) in dogs undergoing treatment (*P* = 0.0274, 0.0020, 0.0128, and 0.0165, respectively). Also, S100A8/A9_USG_, S100A8/A9_Cre_, S100A8/A9_Prot_, S100A12_USG_, S100A12_Cre_, S100A12_Prot_, and uCalR did not differ between dogs treated with a single agent (*n* = 30) compared to combination protocols (*n* = 10; all *P* > 0.05) nor those treated with an NSAID (*n* = 37) compared to dogs not receiving an NSAID (*n* = 3; all *P* > 0.05).

In patients diagnosed with a UTI, S100A8/A9_USG_, S100A8/A9_Cre_, S100A8/A9_Prot_, S100A12_USG_, S100A12_Cre_, S100A12_Prot_, and uCalR did not differ in dogs with a single bacterial isolate (*n* = 43) from those with a polymicrobial UTI (*n* = 7; all *P* > 0.05); and there were no differences seen in dogs where only *Enterococcus* sp. (*n* = 5) and/or other isolates (*n* = 45) were cultured from urine samples (all *P* > 0.05).

### Diagnostic accuracy

The area under the ROC curve (AUROC), optimum cut-off concentrations (and cut-off concentrations for at least a 90% sensitivity and a 90% specificity, respectively), sensitivities, and specificities are summarized in Table 2.

**Table 2:** Sensitivities and specificities at the optimal cut-off values (and cut-offs for resulting in a sensitivity and specificity of at least 90% each), and area under the receiver operating characteristic curve (AUROC) for urinary S100/calgranulins and the S100A8/A9-to-S100A12 ratio (uCalR) to distinguish treatment-naïve dogs with transitional cell carcinoma (TCC)/prostatic carcinoma (PCA) from other groups of dogs (all dogs ≥6 years of age [*n* = 141])

Parameter	AUROC^†^	Cut-off	Sensitivity	Specificity
Treatment-naïve TCC/PCA (*n* = 22) vs. UTI (*n* = 35)				
S100A8/A9_USG_	**0.671**	≥96.3	96%	43%
		≥8180.7	32%	94%
S100A8/A9_Cre_	0.612	≥160.0	96%	35%
S100A12_USG_	0.516	≤3.2	91%	29%
S100A12_Cre_	0.521	≤117.9	68%	62%
uCalR	**0.751**	≥9.1	91%	60%
		≥5.2	100%	49%
		≥72.2	18%	94%
Treatment-naïve TCC/PCA vs. other diseases causing lower urinary tract signs (*n* = 45)				
S100A8/A9_USG_	**0.684**	≥96.3	96%	44%
		≥8230.7	32%	96%
S100A8/A9_Cre_	0.631	≥167.8	96%	39%
S100A12_USG_	0.548	≥7.0	82%	42%
S100A12_Cre_	0.517	≥8.7	96%	34%
uCalR	**0.698**	≥5.2	100%	40%
		≥9.1	91%	49%
		≥72.2	18%	96%
Treatment-naïve TCC/PCA vs. other disease groups (*n* = 86)^a^				
S100A8/A9_USG_	**0.807**	≥109.9	96%	64%
		≥2274.8	36%	92%
S100A8/A9_Cre_	**0.763**	≥188.9	96%	55%
		≥8603.1	27%	91%
S100A12_USG_	**0.712**	≥4.0	91%	57%
		≥776.1	23%	93%
S100A12_Cre_	**0.697**	≥8.7	96%	78%
		≥2538.9	18%	95%
uCalR	**0.664**	≥9.3	91%	42%
		≥83.4	18%	96%
Treatment-naïve TCC/PCA vs. all other groups (*n* = 119)^a^				
S100A8/A9_USG_	**0.824**	≥109.9	96%	66%
		≥2274.8	36%	93%
S100A8/A9_Cre_	**0.787**	≥188.9	96%	59%
		≥5783.2	36%	92%
S100A12_USG_	**0.740**	≥7.0	82%	66%
		≥221.0	27%	90%
S100A12_Cre_	**0.722**	≥8.7	96%	57%
		≥833.8	23%	90%
uCalR	**0.657**	≥9.3	91%	41%
		≥83.4	18%	96%
Treatment-naïve TCC/PCA vs. all other groups, excl. Urinary tract infections (*n* = 84)				
S100A8/A9_USG_	**0.896**	≥109.9	96%	75%
		≥374.9	59%	95%
S100A8/A9_Cre_	**0.875**	≥188.9	96%	70%
		≥792.7	55%	91%
S100A12_USG_	**0.850**	≥8.6	77%	86%
		≥4.0	91%	71%
S100A12_Cre_	**0.838**	≥10.7	91%	76%
		≥92.3	32%	91%
uCalR	0.612	≥9.3	91%	33%

^a^excluding TCC/PCA dogs undergoing anticancer therapy

^†^﻿values in boldface are significant at ﻿*P*﻿ < 0.05

Using a uCalR of ≥9.3 or a S100A8/A9_USG_ of ≥109.9 to screen middle-aged to older dogs (≥ 6 years of age) for TCC/PCA (0.7% estimated prevalence of TCC/PCA) yielded an NPV of 100% (1.000 for both) and a PPV between 1 and 2% (0.011 and 0.019, respectively). If a UTI has been excluded in the same population of dogs (estimated prevalence of TCC/PCA: 1%), a S100A8/A9_USG_ ≥ 109.9 resulted in a NPV of ~100% (1.000) and a PPV of ~4% (0.038). In dogs ≥6 years of age, a uCalR of ≥5.2 [or ≥9.1] or a S100A8/A9_USG_ of ≥96.3 differentiated patients with TCC/PCA from those with non-neoplastic causes of similar presenting signs (estimated prevalence of TCC/PCA in this population: 20%) with a NPV of 96–100% (1.000 [0.956] and 0.975, respectively) and a PPV of 29–31% (0.294 [0.308] and 0.301, respectively).

**Fig. 4:**
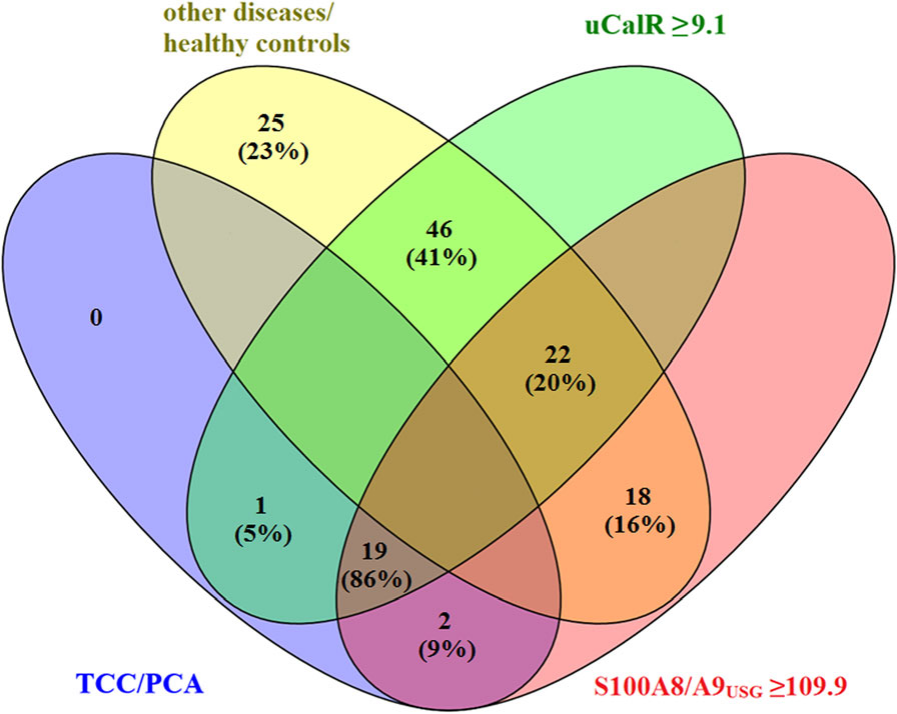
Four-way Venn diagram demonstrating the separation between dogs with TCC/PCA (treatment-naïve) and all other groups of dogs ≥6 years of age by use of a uCalR ≥9.1 combined with a S100A8/A9_USG_ ≥ 109.9

Use of a uCalR of ≥9.1 combined with a S100A8/A9_USG_ of ≥109.9 to screen middle-aged to older dogs (≥ 6 years of age) for TCC/PCA (estimated prevalence: 0.7%) yielded a sensitivity of 86% (95% CI: 67–95%) and a specificity of 80% (95% CI: 72–87%; OR: 25.6, 95% CI: 7.3–88.5) (Fig. 4), with a NPV of ~100% (0.999; 95% Cl: 0.997–1.000) and a PPV of 3% (0.030; 95% CI: 0.020–0.044).

## Discussion

This study evaluated the diagnostic utility of measuring urine concentrations of S100A8/A9 and S100A12 as well as the uCalR in dogs with TCC/PCA (treatment-naïve or undergoing antineoplastic therapy). Measurement of these biomarkers in urine specimens has the advantage of yielding information from the urinary tract. However, the results may be skewed by prerenal factors (e.g., increased amino acid turnover with neoplastic diseases causing glomerular hyperfiltration [35]), renal diseases (e.g., chronic kidney disease), and/or other diseases of the lower urinary tract (e.g., bacterial cystitis or urolithiasis). Thus, dogs with other non-neoplastic diseases of the urinary tract and dogs with neoplastic conditions not involving the urinary tract were included as disease control groups of dogs.

Diagnostic accuracy of the urinary canine S100/calgranulins was evaluated in dogs ≥6 years of age to mimic the situation of using these biomarkers in the clinical setting to screen for or diagnose dogs with TCC/PCA. The results of this study showed that urine S100A8/A9 and the uCalR may have limited diagnostic utility in dogs with TCC/PCA. Concentrations of urine S100A8/A9 as well as the parameters of diagnostic accuracy (AUROC, sensitivity, and specificity) and the optimum cut-offs for the separation of dogs diagnosed with TCC/PCA from the other groups of dogs were comparable to those in one study evaluating urine S100A8/A9 concentrations in humans with bladder cancer [30]. The study by Ebbing et al. also showed higher urine concentrations of S100A8/A9 in people with higher grade urothelial carcinomas [30]. However, the possibility of a correlation between urinary concentrations of the S100/calgranulins and the results of complete cancer staging and/or grading, response to treatment, and the individual patient outcome were not investigated as part of our study, and this will need to be further explored. Further research is also needed to determine when changes in S100A8/A9 and S100A12 could possibly occur along the spectrum of tumor development and whether these tests can detect pre-cancerous pathology or early cancer.

The components of diagnostic accuracy for the urinary S100/calgranulins in this study were higher than the sensitivity and specificity reported for urine sediment analysis, where neoplastic cells are detectable in approximately 30% of patients with TCC but neoplastic cells can be difficult to distinguish from reactive epithelial cells associated with inflammation [3, 5]. Also, both sensitivity and specificity of the S100A8/A9_USG_ were higher than those of the V-BTA [6–9]. But similar to the V-BTA measurement, the S100A8/A9_USG_ in dogs ≥6 years of age also suffers from a moderate rate of false positives (59% for dogs with a UTI and 24% [18–33%] for the remaining groups of dogs when using a cut-off of ≥109.9), which is also consistent with the increased urinary proteome fraction of S100A8/A9 and S100A12 (i.e., S100A8/A9_Prot_ and S100A12_Prot_) in TCC/PCA dogs with concurrent UTI. An additional advantage over the V-BTA test [8], S100A8/A9_USG_ is not affected by hematuria and offers a quantitative result [34].

The high sensitivity and NPV of S100A8/A9_USG_ observed in this study suggest that measuring S100A8/A9_USG_ could be a good screening test for TCC/PCA in dogs, especially patients where a UTI has been ruled out as a cause of clinical signs of lower urinary tract disease, and that a negative test result (i.e., a S100A8/A9_USG_ of <109.9) essentially excludes a diagnosis of TCC/PCA in dogs. The uCalR, on the other hand, appears to be a better marker to distinguish patients with a UTI from those with TCC/PCA. However, the false negative rate of the uCalR in dogs ≥6 years old with a UTI was still moderate (54% and 43% when using ≥5.2 and ≥9.1 as the cut-off concentration, respectively), but a combination of S100A8/A9_USG_ and the uCalR improved the diagnostic accuracy (i.e., specificity) for the detection of TCC/PCA in dogs. Similar to the V-BTA test [8], the PPV of the uCalR and S100A8/A9_USG_ increased to about 30% if used in a population of dogs with a higher index of suspicion for lower urinary tract disease. Further prospective studies are warranted in a larger cohort of dogs to validate our findings and to directly compare the diagnostic performance of the urinary S100/calgranulins with the V-BTA test.

Dog breeds reported to have a higher risk of developing TCC (Scottish Terrier, West Highland White Terrier, Shetland Sheepdog, Beagle, and Dachshund) [1–3, 36] were overrepresented (36%) in our study. The female:male ratio (1.4:1) as well as the urinary bladder trigone being the most common tumor site were similar to previous reports [1, 3, 4, 8]. Detection of a concurrent UTI in 42% of the dogs with TCC/PCA (both treatment-naïve dogs and dogs undergoing anticancer therapy) and the spectrum of organisms isolated are also in line with the results of others [37].

An interesting finding of the current study is that, compared to dogs with TCC/PCA that received anticancer treatment at the time of urine sample collection, treatment-naïve dogs with TCC/PCA had 2.1- to 2.5-fold higher urinary S100A8/A9 concentrations and 2.5- to 2.9-fold higher urinary S100A12 concentrations (albeit statistical significance was reached only for S100A12_Cre_). While the cause of urinary S100/calgranulins being lower in dogs with TCC/PCA under treatment cannot be evaluated through this study, potential explanations include a decreased expression of these proteins by tumor cells or reduction of tumor-associated inflammation (particularly tumor-associated or even metastasis-associated macrophages) in response to treatment [38]. However, with the source and treatment-induced change of S100A8/A9 and S100A12 expression in canine TCC/PCA being unknown and the relatively large variation in the uCalR seen in the group of TCC/PCA dogs receiving anticancer treatment, it remains to be determined whether the S100/calgranulins are part of a pro-tumorigenic [39] or anti-tumorigenic [40] signature in canine TCC/PCA [38], or if they may even have a dual role. This distinction is particularly important as the synergism and convergence in the signaling pathways of inflammation and cancer – especially the release of damage-associated molecular pattern molecules (such as S100A8/A9 and S100A12), downstream activation of nuclear factor-κB, and expression of inflammatory cytokines and chemokines – can lead to positive feedback loops perpetuating chronic inflammation and thus a pro-tumorigenic microenvironment [13, 38].

From a clinical perspective, these aspects lend themselves to research of novel molecular-based therapeutic strategies aimed at targeting tumor-associated inflammation, which can drive tumor progression and metastasis [38, 41]. From a comparative oncology standpoint, study of the S100/calgranulins in canine TCC/PCA is of very high interest because here the spontaneous disease in dogs serves as a better model for human urothelial carcinoma than murine models (rodents lack S100A12 and S100A8 appears to functionally resemble S100A12 [42–44]).

Another interesting finding in TCC/PCA dogs receiving anticancer therapy is that urinary S100A8/A9 and S100A12 concentrations were lower with primary involvement of the urinary bladder than that of the prostate. Whether this reflects a higher degree of tumor-associated inflammation with prostatic involvement, the response (increased antitumor immunity or treatment-induced inflammation) or even lack of response to treatment [38] requires further study. In view of this finding, the lack of a difference in urinary S100/calgranulin concentrations between TCC/PCA dogs given an NSAID (either alone or as part of a combination protocol) and those receiving other forms of antineoplastic therapy was unexpected as metronomic NSAIDs predominantly affect the tumor-promoting inflammation [1, 38] and the expression of S100A8 and S100A9 has been indirectly linked to the cyclooxygenase (COX)-2/cAMP pathway [45, 46].

We acknowledge the limitation of the study that healthy control dogs did not undergo the same diagnostic evaluation as patients with TCC/PCA or other urinary tract or non-urinary tract diseases. Thus, the possibility of an occult urinary tract disease (including TCC/PCA) cannot be excluded with certainty in this group of dogs.

## Conclusions

The results of this study show that urine S100A8/A9 and the uCalR have utility as an exclusionary tool in dogs with suspected TCC/PCA. Despite a limited diagnostic value (i.e., as a confirmatory test), the high sensitivity and NPV of S100A8/A9_USG_ suggests that S100A8/A9 could be a good screening test for TCC/PCA in dogs and that the uCalR can help differentiate dogs with a UTI. Further studies are warranted to validate our findings in a larger cohort of dogs, to evaluate the source of S100A8/A9 and S100A12 expression in canine TCC/PCA patients, and to determine whether S100A8/A9 and/or S100A12 expression correlates with tumor grade and/or complete tumor stage, response to treatment, progression of cancer, and/or survival time.

## Abbreviations

AUROC: Area under the ROC curve
CI: Confidence interval
NPV: Negative predictive value
PCA: Prostatic carcinoma
PPV: Positive predictive value
ROC: Receiver operating characteristic curve
S100A12: S100A12 protein
S100A8/A9: S100A8/A9 (calprotectin) protein complex
TCC: Transitional cell carcinoma
uCalR: Urine S100A8/A9-to-S100A12 ratio
USG: Urine specific gravity
UTI: Urinary tract infection

## Footnotes

1 Clinical refractometer, Atago USA Inc., Bellevue, WA, USA.

2 Sirrus Creatinine LiquiColor, Stanbio Laboratory, Boerne, TX, USA.

3 Coomassie protein Bradford assay, Thermo Fisher Scientific Inc., Rockford, IL, USA.

4 Analyse-it® v4.20.1 Method validation edition, Analyse-it Software, Leeds, West Yorkshire, UK.

5 JMP® v12.0, SAS Institute Inc., Cary, NC, USA.
